# Authentication of protein, fat, carbohydrates, and total energy in commercialized high protein sports foods with their labeling data

**DOI:** 10.1038/s41598-023-42084-3

**Published:** 2023-09-16

**Authors:** Maged Ossama Aly, Somia Mohamed Ghobashy, Samar Mohamed Aborhyem

**Affiliations:** 1 Nutrition Department, High Institute of Public Health, Alexandria University, 165 El-Horreya Ave. El-Hadara, Alexandria, Egypt; 2 Central Laboratories, High Institute of Public Health, Alexandria University, Alexandria, Egypt; 3 Nutrition Department, High Institute of Public Health, Alexandria University, Alexandria, Egypt

**Keywords:** Nutrition, Public health

## Abstract

The popularity of high-protein sports food items among athletes and the bodybuilding community has risen dramatically. This study aimed to authenticate the reported per serving food label content of protein, fat, carbohydrate, and total energy in commercially available high-protein sports foods available in Egyptian markets. A cross-sectional study was performed on a total of forty-five samples of nine products that included protein bars, cookies, vegan bars, puffs, whey protein, protein drinks, peanut butter, pancake mix, and Greek yogurt. Protein and fat analysis were done according to Kheldahl and Folich methods, respectively, while carbohydrate was calculated by difference. Total energy was calculated according to their content. A significant (*p* < 0.001) difference was found between the laboratory-assessed content and the reported food label values in protein, carbohydrate, and energy. Protein sport food products had significantly lower protein content (11.6 ± 4.67) obtained from laboratory measurement than the label reported value (17.17 ± 7.22). The fat content in vegan protein was 149.3% higher than the label values (1.67 vs. 0.67 g/serving). The mean fat content per serving of 30 out of 45 samples was significantly higher than the food label values in the bar (37.8%), puffs (32.7%), vegan protein (149.3%), and protein drinks (28.6%). These differences may result in compromised performance and undesired fat gain, as opposed to a desired increase in muscle mass, which could compromise the desired impact of the consumed sports foods.

## Introduction

Nutrition and physical activity are two essential components of a healthy lifestyle. Eating a balanced diet and engaging in regular physical activity are both important for maintaining good health^1,2^. Sports nutrition is a rapidly growing field, and there is an increasing focus on the importance of personalized nutrition in sports performance. Different sports require different types of nutrition, and athletes should tailor their diets to meet their individual needs^3^. In addition to the normal diet, some sports disciplines may require the consumption of dietary supplements to improve performance^4,5^. However, it is important to note that dietary supplements should not be used as a substitute for a healthy diet. Supplements should only be used if recommended by a healthcare professional, and athletes should be aware of the potential risks associated with taking supplements^6^.

Currently, there is a multitude of competing products with health and performance enhancement claims, such as sports foods, dietary supplements, and fortified and functional foods, which have some overlap or shared characteristics. Therefore, the Australian Institute of Sport (AIS) provided a working definition to compare these products:^7^

Supplements are single or multi-ingredient products in powder, limited-volume liquid, pill, or capsule form providing nutrients or other dietary components to achieve a specific health and/or performance benefit. Sports Food: food or drink formulated to help people achieve specific nutritional or performance goals by providing a convenient form of general nutrition support for athletes or a targeted use around exercise (e.g., liquid meal replacements) or for targeted use around exercise (e.g., sports drinks, gels, bars).

Fortified food is a conventional food to which vitamins or other nutrients are added during processing to increase its nutritional value, while functional food is foods enriched with additional nutrients or components outside their typical nutrient composition for the purpose of enhancing the functional properties of the inherent nutrient profile of the food.

The increase in public awareness of sports has been a major factor in the development of the sports food industry. As more people become aware of the importance of physical activity and the benefits of a healthy lifestyle, they are more likely to invest in sports foods to meet their nutritional needs. Sports foods provide the necessary nutrients to meet the metabolic and energy needs of athletes and active individuals^8^. Currently, there are various forms of sports foods available in the market, including drinks, solid foods, semi-solid foods, and protein powder supplements. Solid sports foods are usually bars or snacks that provide carbohydrates, proteins, and fats. Drinks and semi-solid sports foods are usually shakes or gels that provide carbohydrates and proteins^9^. Athletes consume or are advised to consume fluids and electrolytes (sodium) for a variety of reasons before, during, and after exercise. For the most part, these factors aim to maintain total body hydration because deficits (hypohydration) will worsen aerobic performance and increase thermal and cardiovascular strain. Sweat, which contains both water and electrolytes, is produced during vigorous exertion and in warm/hot temperatures. Active athletes' daily water (4–10 L) and sodium (3500–7000 mg) losses during hot weather exposure might result in dehydration and electrolyte deficiencies. To reestablish euhydration status, both water and sodium must be replenished^10^. Therefore, electrolyte replacement supplements, powders, tablets, or ready-to-drink forms, have good evidence for use to promote effective rehydration strategies and restore fluid and electrolytes (particularly sodium and potassium) lost through perspiration or other bodily fluids^11^.

The expanding market for sports foods has created a greater demand for these products. Additionally, advances in manufacturing technology have allowed for the production of more efficient and cost-effective sports foods. Finally, increasing funds investment in the sports food industry has allowed for more research and development of new products^8^.

Proteins are essential for muscle growth and repair, and they are also important for maintaining strength and preventing nutritional deficiencies such as Protein-energy malnutrition (PEM)^12,13^. Protein-enhanced food products are an important part of the sports industry, and the marketing of these products is carefully planned. Additionally, they are the most popular type of ergogenic aid, accounting for 80% of sports nutrition sales. These products are usually found in bars, powder, milk, yogurt, and other food forms, although functional protein beverages are becoming increasingly popular. Moreover, protein supplementation is a major nutritional practice among professional and amateur athletes, representing a market of $5 billion in the USA alone^14–16^. The classification of specialized foods for athletes needs scientific justification, and the most reasoned approach for ranking foods for athletes is proposed by the European Commission's Scientific Committee on Nutrition. This approach takes into account the analysis of the macronutrient composition and energy value of specialized foods, as described in Table 1.

**Table 1:** Nutritional and energy value of sports food products of different categories^17^.

Product category	Content	Intervals (%)	Energy	value/calorie Content (kcal in 100 g)
	Protein	Fat	CHO	
Category A (foods rich in carbohydrates)	5–50	0–5	50–95	220–425
Category B (carbohydrate-electrolyte solutions)	0	0	3–8	12–32
Category C (proteins and their components)	50–90	0–5	4–50	216–425
Category D (vitamin and mineral complexes, as well as sources of minor and essential compounds)	0	0–1	0	0–9

Daher and colleagues, in their review, reported that the prevalence of dietary supplement use among athletes worldwide ranged from 11 to 100%, and the most commonly used supplements were protein and vitamin/mineral supplements^18^. The promotion of nutritional products for athletes is often focused on the idea of a "healthy lifestyle," "proper nutrition," and "normalization of metabolism and body functions." This type of marketing is designed to appeal to athletes who are looking to improve their performance and health^19^. Companies use this type of messaging to emphasize the importance of proper nutrition and the benefits of using their products. As a result, the global functional food market was expected to exceed 300 billion USD by 2020, with sports nutrition products playing a major role in this growth^20^. Therefore, the purpose of this study was to analyze the content of high-protein sports food products, including carbohydrates, fats, and protein, and calculate the total calories as well as authenticate them with their nutritional labels.

## Material and methods

### Part I: chemical analysis

An experimental study was conducted, and a total of forty-five different available high protein products were purchased from sports gyms, Amazon websites, or hypermarkets according to their availability and their high consumption rate, depending on a previous pilot study. Newly produced products with valid expiry dates were used in this study. Samples were stored at room temperature, except dairy products were stored in a refrigerator at 4ºC until analysis. Samples were ground well before analysis. All the following tests were performed in triplicates.

### Proximate analysis

Moisture content and total ash were determined according to the Association of Official Agricultural Chemists (AOAC) (2000), while total protein and fat content were determined by the conventional Khjeldahl and Folch methods, respectively. Total carbohydrates were calculated by difference according to the AOAC method^21–23^

### Protein quantification according to the Khjeldahl method

Protein and other organic food components in the sample were digested with sulfuric acid in the presence of catalysts. The total nitrogen is converted to ammonium sulfate. The digest was neutralized with alkali and distilled into a boric acid solution. The borate anions formed are titrated with standardized hydrochloric acid, which is converted to nitrogen in the sample. The result of the analysis represents the nitrogen content of the food since nitrogen also comes from non-protein components such as free amino acids, small peptides, nucleic acids, phospholipids, amino sugars, porphyrin, and some vitamins, alkaloids, uric acid, urea, and ammonium ions. Hence results should be multiplied by a factor to be converted to crude protein according to AOAC in methods 976.06, 976.05, and 960.52 (2000) method^21^.

Calculation

$$\% {\text{ crude protein }} = { 6}.{25} \times \% {\text{N}}$$

$$\% {\text{N }} = \, \left( {{\text{S}} - {\text{B}}} \right) \times {\text{N}} \times 0.0{\text{14D}} \times {1}00,\;{\text{Wt}}.{\text{ of the sample}} \times {\text{V}}$$

where

*S* = Sample titration reading.

*B* = Blank titration reading.

*N* = Normality of HCl.

*D* = Dilution of the sample after digestion.

*V* = Volume taken for distillation.

0.14 Milli equivalent weight of nitrogen

### Fat quantification according to Folch method

Samples were mixed with methanol and chloroform in such proportions as to give a single phase miscible with water. Additional chloroform is then added to give a separation of phases; the solvents are separated by centrifugation. The chloroform layer contains the dissolved fat, which is left to evaporate. The extracted fat residue is weighted^22^.

Calculation

$$\% {\text{ Crude fat }} = {\text{ wt of fat}} \times {1}00/{\text{wt of sample}}$$

### Moisture quantification %

Moisture is the measurement of weight loss due to the evaporation of water at or near boiling point. Moisture was measured according to the Association of Official Analysis Chemists (AOAC) (2000) method 990.19^21^.

Calculation

$${\text{Moisture }}\% \, = \, \left( {{\text{W}}_{{1}} - {\text{W}}_{{2}} } \right) \times {1}00/{\text{wt of sample in grams}}$$

where

$${\text{W}}_{{1}} = {\text{weight of crucible }} + {\text{ sample before drying}}.$$

$${\text{W}}_{{2}} = {\text{weight of crucible }} + {\text{ sample after drying}}.$$

### Ash quantification % according to AOAC

Dry ashing refers to the use of a muffle furnace capable of maintaining a temperature of 500 °C–600°C. Water and volatiles are vaporized, and organic substances are burned in the presence of oxygen to CO_2_ and oxides of N_2_ according to AOAC 900.02 A (2000) method as follows^21^:

Calculation

$${\text{Ash }}\% \, = {\text{ Difference in weight of fluffy ash}} \times {1}00/{\text{Wt}}.{\text{ of sample in grams }}\left( {{\text{W}}_{{2}} } \right)$$

$${\text{Difference in weight of ash }} = {\text{ W}}_{{3}} - {\text{W}}_{{1}}$$

The percentage of ash was calculated (dry weight basis) as follows:

$${\text{Ash }}\% \, = {\text{ Difference in weight of fluffy ash}} \times {1}00/{\text{Wt}}.{\text{ of sample in grams }}$$

The percentage of ash was calculated (wet weight basis) as follows:

$${\text{Ash }}\%_{{({\text{wet basis}})}} = \frac{{\left( {{\text{wt}}.{\text{ crucible and ash }} - {\text{ wt}}.{\text{ crucible}}} \right) }}{{\left( {{\text{wt}}.{\text{ crucible and sample }} - {\text{ wt}}.{\text{ crucible}}} \right)}} \; \times \;{1}00$$

### Carbohydrate % according to AOAC

Carbohydrate content was calculated according to AOAC (2000) method using the following equation^21^:

$${\text{Carbohydrates }}\% \, = { 1}00 \, {-} \, \left( {{\text{Protein }}\% \, + {\text{ Fat }}\% \, + {\text{ Ash }}\% \, + {\text{ Moisture }}\% } \right)$$

Caloric Content^23^

Caloric conversion information on the label for fat, carbohydrate, and protein is calculated by the following equation.

$${\text{Total}}\;{\text{calories}}\;\left( {{\text{cal}}} \right)\, = \,\left( {{4}\, \times \,{\text{protein}}\;{\text{in}}\;{\text{grams}}} \right)\, + \,\left( {{4}\, \times \,{\text{carbohydrates}}\;{\text{in}}\;{\text{grams}}} \right)\, + \,\left( {{9}\, \times \,{\text{fats}}\;{\text{in}}\;{\text{gram}}} \right).$$

### Ethical approval

The researchers got the approval of the Ethical Committee, High Institute of Public Health, Alexandria University, and it complies with the International Guidelines for Research Ethics. There is no conflict of interest.

### Data analysis

Data were fed to the computer and analyzed using IBM SPSS software package version 20.0. (Armonk, NY: IBM Corp). For continuous data, they were tested for normality by the Shapiro–Wilk test. Quantitative data were expressed as a range (minimum and maximum), mean, and standard deviation for normally distributed quantitative variables Paired t-test was used to compare nutritional facts per serving and laboratory nutritional facts per serving. On the other hand, for not normally distributed quantitative variables, Wilcoxon signed ranks test was used to compare nutritional facts per serving and laboratory nutritional facts per serving. The significance of the obtained results was judged at the 5% level^24,25^.

## Results

Data in Table 2 reported that there is a significant change (*p* < 0.001) among nutritional facts (protein, carbohydrate, and energy) between labeling data and laboratory measurements. Different studied protein sport foods products recorded a significant decrease in their protein content on comparing nutritional facts obtained from laboratory measurement and labeling data to be 11.6 ± 4.67 and 17.17 ± 7.22 respectively, while there is a significant increase in CHO as follows 29.26 ± 15.98 and 19.45 + _19.54 ± 17.48 respectively. On the other hand, there is no significant change in fat content.

**Table 2:** Comparison between nutritional facts per serving and laboratory nutritional facts per serving and per 100 g according to energy, protein, CHO, and fat (*n* = 45).

	Labeled Nutritional facts per serving	Laboratory nutritional facts per serving	Laboratory nutritional facts per 100 g	Test of Sig.	*P*
Energy (kcal)					
Min.–Max	100.0–385.0	99.0–388.0	66.0–614.0	t = 3.642*	0.001*
Mean ± SD	197.38 ± 58.33	227.09 ± 71.68	388 ± 57.69		
Protein (g)					
Min.–Max	7.0–33.0	2.0–24.0	5.0–63.0	Z = 4.920*	< 0.001*
Mean ± SD	17.17 ± 7.22	11.60 ± 4.67	20.0 ± 13.24		
CHO (g)					
Min.–Max	4.0–100.0	5.0–72.0	3.0–77.0	Z = 4.607*	< 0.001*
Mean ± SD	19.54 ± 17.48	29.60 ± 15.98	48 ± 21.67		
Fat (g)					
Min.–Max	0.0–15.0	0.0–15.0	0.0–44.0	Z = 1.351	0.177
Mean ± SD	6.29 ± 3.90	6.93 ± 3.49	13.0 ± 9.9		

*SD*: Standard deviation t: Paired *t*-test Z: Wilcoxon signed ranks test.

*p*: *p*-value for comparing nutritional facts per serving and laboratory nutritional facts per serving.

*Statistically significant at *p* ≤ 0.05.

Pancake mix: Excluded from the comparison due to the small number of cases (*n* = 1).

^#^: Significant with Protein bar.

Results obtained in Table 3 showed that protein cookies had the highest energy (244.67 kcal) followed by protein bar products (220.93 kcal), then protein puffs according to labeling facts (201.78 kcal), which are not matched with measured data in the laboratory, protein cookies had the highest protein content 270 g followed by protein puffs 265.57 g, protein bars 265.11 g, and vegan protein bars 236 g. The labeled energy content (Kcal.) of cookies (244.67 kcal.) is lower than laboratory measurements (270 kcal.) by 9.95%, while labeled nutritional fact regarding vegan protein (132.67 kcal.) is lower than laboratory measurements (143 kcal.) by 7.6% when compared to labeled data.

**Table 3:** Comparison between labeled nutritional facts per serving and laboratory nutritional facts per serving according to energy, protein, carbohydrate, and fat (*n* = 45).

	Type	Number of samples	Labeled nutritional facts per serving	Laboratory nutritional facts per serving	% Change from labeling data
Energy (kcal)	Protein bar	14	220.93 ± 37.8	265.57 ± 37	↑ 24.54
	Vegan protein bar	4	213.25 ± 35.4	236.0 ± 52	↑ 10.14
	Protein cookie	3	244.67 ± 25	270.0 ± 41	↑ 9.95
	Pancake mix	1	150.0 ± 0	178.0 ± 0	↑ 18.67
	Protein puffs	9	201.78 ± 92	265.11 ± 91	↑ 43.11
	Vegan protein	3	132.67 ± 26	143.0 ± 31	↑ 7.6
	Peanut butter	5	192.60 ± 16	184.0 ± 14	↓ − 3.94
	Protein Drinks	2	197.50 ± 15	189.0 ± 20	↓ − 4.41
	Greek yogurt	4	120.0 ± 19	114.0 ± 11	↓ − 4.13
Protein (g)	Protein bar	14	22.50 ± 5.8	11.71 ± 4	↓ − 46.4
	Vegan protein bar	4	13.0 ± 4.8	12.25 ± 1	↓ − 1.35#
	Protein cookie	3	15.67 ± 4	11.0 ± 1	↓ − 27.22
	Pancake mix	1	18.0 ± 0	15.0 ± 0	↓ − 16.67
	Protein puffs	9	13.67 ± 8	7.44 ± 4	↓ − 40.3
	Vegan protein	3	26.0 ± 7	21.67 ± 2	↓ − 14.76
	Peanut butter	5	11.40 ± 2	9.20 ± 2	↓ − 17.48
	Protein Drinks	2	17.50 ± 4	18.0 ± 1	↑ 5.83
	Greek yogurt	4	11.88 ± 2	11.75 ± 4	↓ − 2.14#
CHO (g)	Protein bar	14	19.86 ± 10.6	38.07 ± 6	↑ 231.15
	Vegan protein bar	4	20.25 ± 10.1	28.75 ± 10	↑ 65.63
	Protein cookie	3	22.0 ± 5	35.0 ± 11	↑ 58
	Pancake mix	1	15.0 ± 0	24.0 ± 0	↑ 60
	Protein puffs	9	34.0 ± 31	43.22 ± 19	↑ 95.69
	Vegan protein	3	5.0 ± 2	11.0 ± 3	↑ 142.86
	Peanut butter	5	7.20 ± 1	10.80 ± 3	↑ 53.65
	Protein Drinks	2	24.0 ± 8	20.0 ± 6	↓ − 15.56
	Greek yogurt	4	8.55 ± 2	9.75 ± 3	↑ 16.13
Fat (g)	Protein bar	14	5.29 ± 2.1	7.29 ± 2	↑ 68.25
	Vegan protein bar	4	8.50 ± 1.9	8.25 ± 2	↓ − 1.04
	Protein cookie	3	11.0 ± 3	10.0 ± 4	↓ − 10.47
	Pancake mix	1	2.0 ± 0	2.0 ± 0	0.0
	Protein puffs	9	5.11 ± 2	6.78 ± 3	↑ 48.32
	Vegan protein	3	0.67 ± 1	1.67 ± 1	0.0
	Peanut butter	5	13.60 ± 2	11.60 ± 2	↓ − 14.1
	Protein Drinks	2	3.50 ± 1	4.50 ± 1	↑ 29.17
	Greek yogurt	4	4.30 ± 2	3.0 ± 1	↓ − 25.25

Data were expressed using Mean.

Pancake mix: Excluded from the comparison due to the small number of cases (*n* = 1).

^#^: Significant with Protein bar.

Percent change of energy among nine different sport food products increased in six products, and the highest % increase was recorded in protein puffs 43.11% then protein bars 24.54%, and pancake mix 18.67%, while the lowest % increase was in vegan protein by ↑7.6%. On the other hand, the measured energy (kcal) was lower than the labeled data in Greek yogurt (4.11% lower), peanut butter (3.64% lower), and protein drinks (4.41% lower).

Protein drinks are the only product among nine different types that has laboratory protein content higher than labeled data by 5.83%, protein bars had lower protein content than labeled facts by 46.4%, followed by protein puffs (40.3%), then protein cookies products (27.22). There is no significance in percent change among all tested samples regarding; energy (Kcal), fat(g), and CHO (g). On the other hand, a significant change was recorded among Greek yogurt (↓2.14%) and vegan protein products (↓1.35%).

There is a wide variation regarding carbohydrate content gram/serving on comparing measured laboratory results with labeled data by 142.86% in vegan protein, 231.15% in protein bar, 60% in pancake mix, 58% in protein cookies, and 65.63% increase in vegan protein bar. On the other hand, protein drinks are the only category that had a lower carbohydrate content than labeled data by 15.56%↓. Peanut butter had the lowest carbohydrate/ serving according to laboratory results among different sport food products, while vegan protein reported the lowest content according to labeled data, 10.8 and 5 g/serving. Protein puffs had the highest content in laboratory results and labeled data 43.22 and 34 g/serving.

Fat content in pancake mix is the same in labeling data and measured results in the laboratory. Otherwise, the fat content in the protein bar was higher by 68.25% than the labeling data (7.29 and 5.29 g/serving, respectively), followed by protein puffs by 48.82%, while fat content in Greek yogurt recorded lower results compared to labeling data by 25.25% followed by peanut butter (14.1%) then protein cookies (11 g/serving). Nutritional facts concerning fat content (gram/ serving) in the nine different protein food products were nearly matched in laboratory results and labeling data. Peanut butter had the highest fat (11.6 and 13.6), followed by protein cookies (10 and 11), vegan protein bars (8.25 and 8.5) then protein bars (7.29 and 5.29), while pancake mix had the lowest fat content (2 g/serving).

**Figure 1:**
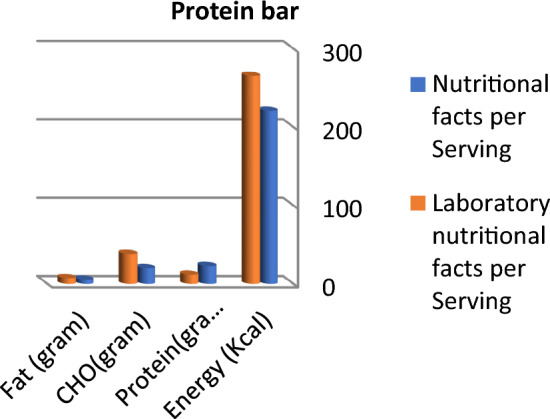
Comparison between labeled nutritional facts and laboratory nutritional facts per serving in protein bar products (*n* = 14) Calculated energy (kcal), fat, and carbohydrate in grams are higher than labeled data in protein bar products; otherwise protein content is lower than labeled facts.

**Figure 2:**
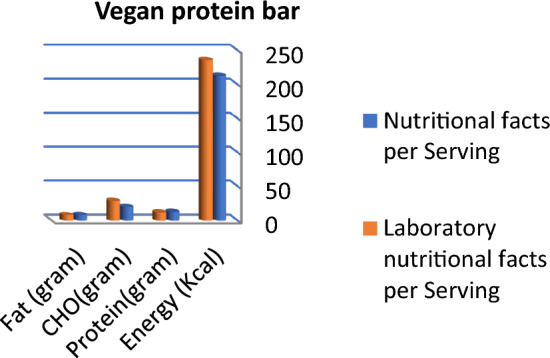
Comparison between labeled nutritional facts and laboratory nutritional facts per serving in vegan protein bar products (*n* = 4) Protein and fat content in vegan protein bars are nearly the same in labeled facts and laboratory results, while lab. Results concerning carbohydrates and total energy were higher than labeled facts.

**Figure 3:**
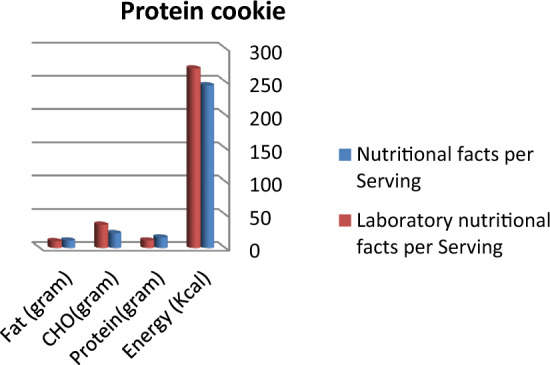
Comparison between labeled nutritional facts and laboratory nutritional facts per serving in protein cookie products (*n* = 3) Labeled nutritional facts concerning protein and fat content in cookie products record higher content than laboratory results, while energy and carbohydrate records lower data.

**Figure 4:**
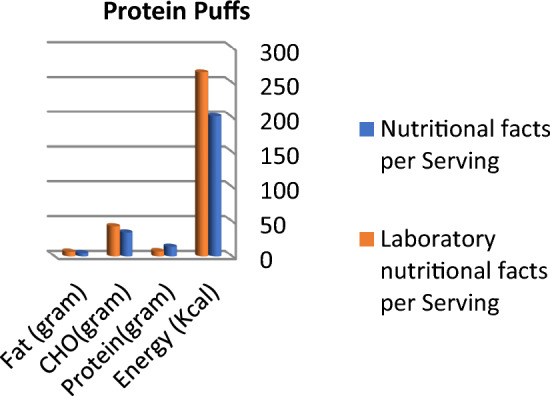
Comparison between labeled nutritional facts and laboratory nutritional facts per serving in protein puffs products (*n* = 9) Protein content was lower than labeled data, while laboratory results were higher than labeled facts in energy and carbohydrate content.

**Figure 5:**
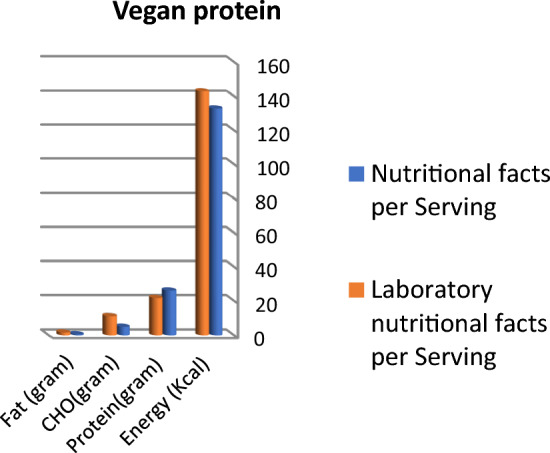
Comparison between labeled nutritional facts and laboratory nutritional facts per serving in vegan protein products (*n* = 3) Labeled nutritional facts in vegan protein record higher protein content than laboratory results, while other nutritional facts ( fat, Cho, and energy) record lower content.

**Figure 6:**
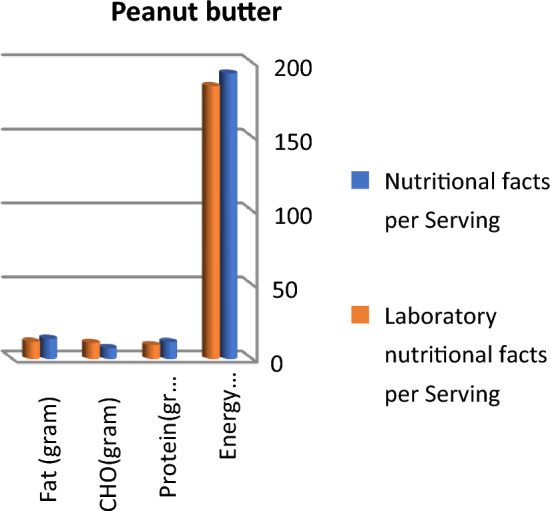
Comparison between labeled nutritional facts and laboratory nutritional facts per serving in peanut butter products (*n* = 5) Labeled nutritional facts (energy, fat, and protein in peanut butter products were higher than laboratory results per serving.

**Figure 7:**
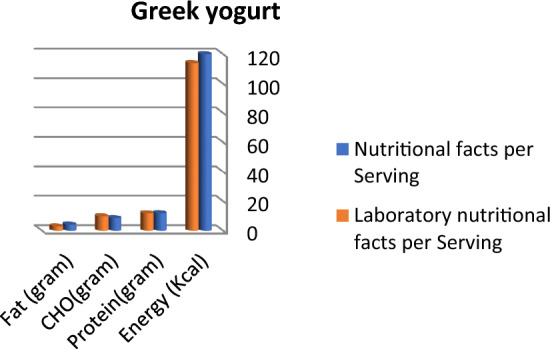
Comparison between labeled nutritional facts and laboratory nutritional facts per serving in Greek yogurt products (*n* = 4) There is a slight change in labeled facts and laboratory results among protein and carbohydrate content in Greek yogurt products, otherwise energy and fat content records higher concentrations in labeled facts.

Calculated energy (kcal), fat, and carbohydrate in grams are higher than labeled data in protein bar products; otherwise, protein content is lower than labeled facts, as presented in Fig. 1. Protein and fat content in vegan protein bars are nearly the same in labeled facts and laboratory results, whereas lab. results concerning carbohydrates and total energy were higher than labeled facts (Fig. 2). Labeled nutritional facts concerning protein and fat content in cookie products record higher content than laboratory results, while energy and carbohydrate records lower data, as shown in Fig. 3. Protein content was lower than labeled data, while laboratory results were higher than labeled facts in energy and carbohydrate content (Fig. 4). The labeled nutritional facts in vegan protein record higher protein content than laboratory results, while other nutritional facts (fat, Carbohydrates, and energy) record lower content (Fig. 5). Labeled nutritional facts (energy, fat, and protein in peanut butter products were higher than laboratory results per serving. Labeled nutritional facts (energy, fat, and protein in peanut butter products were higher than laboratory results per serving (Fig. 6). On the other hand, data in Fig. 7 showed that there is a slight change in labeled facts and laboratory results among protein and carbohydrate content in Greek yogurt products; otherwise, energy and fat content records higher concentrations in labeled facts.

## Discussion

Food labels may be causing more harm than good. Food labels' intentions may be worthy of promoting healthy eating, although they may be very misleading claims and incorrect nutritional facts^26^. Because neither these claims nor ingredients are reviewed by the US Food and Drug Administration (FDA) and are confusing for customers"^27^. Since Dietary supplements are considered a food subcategory under the Dietary Supplement Health and Education Act of 1994 (DSHEA), they do not require premarket approval by the FDA^28^.

Supplements are compounds used in sports to raise energy, enhance performance, and prevent nutritional shortages. In terms of sports supplementing, protein supplements have proven to be an intriguing strategy. This is because it has a wide range of advantages, including improved body composition, lean muscle or hypertrophy, and enhanced strength, endurance, and physical performance^29^. A study was conducted to assess the prevalence of supplement usage among athletes in Alexandria, Egypt; it concluded that 42% of the athletes use dietary supplements, and the most common type of supplements used by athletes was protein supplements (67.9%)^30^. The utilization of high-protein products is becoming ever more common among amateur and professional athletes. Both national and international consumption has risen recently^31^. Due to its nutritious value, people are paying more attention to it. In order to prevent related hazards and maximize the benefits of supplement intake, the hypothesis is proposed that it is vital to evaluate the proximate analysis of these supplements in accordance with the information provided on their labels^32^. The present data revealed that there is a significant change between labeled nutritional facts per serving and laboratory nutritional data per serving (*p* < 0.001^*^) regarding protein, energy, and carbohydrates.

Despite the lack of a uniform consumption policy, the International Olympic Committee and the International Society of Sports Nutrition advise combining resistance training with a daily protein intake of 1.6–2.2 g/kg of body mass/day, which has been found to be approximately 25–30 g/mean to optimize the anabolic use of protein. This figure may be thought of in terms of daily total protein consumption^12,33,34^. Even though the market's availability of high-protein bars, most of them include protein ingredients (20–50 g of high-quality protein per 100 g of the product)^35^, which agreed with some items in our studied samples and disagreed with others. That confirms the inaccurate labeling data with the laboratory results.

Regarding energy content among 45 different high protein products, three different products with a total of 11 samples had lower total calculated energy (kcal.) from obtained laboratory results than labeled data (peanut butter, protein drinks, and Greek yogurt products); otherwise, the rest of studied samples of a total 34 samples had higher total energy (kcal.) than labeled data which indicates the inaccurate information of nutritional facts. Gym users mainly rely on protein supplements for muscle building and to improve their physical activity according to a study done to assess knowledge, attitudes, and use of protein supplements among Saudi adults concluded that 48.3% of the participants from Saudi adults agreed that high protein products necessary for muscle building through weight lifting. In addition, 44.2% of the participant confirmed that protein supplements are a good source of energy during workouts^36^. Gym users and athletes consume high protein products mainly to gain their requirements and to help them either build mass muscle or/ and improve their physical performance. Results in Table 3 illustrated that all studied products recorded lower protein content than labeled data except protein drinks brands had higher content than labeled information by 2.9%. These data may reflect adulteration and inaccuracy of labeling data that affect the main purpose of their consumption to help in improving physical performance, building muscle mass, and aiding in weight loss. These findings are harmonized with a study done in Saudi Arabia^36^ concluded that 48% of participants said that their consumption of protein supplements and high-protein products helps them in building muscle mass. However, 14.55% don't know the benefit of these products.

The question here is how come the calculated energy from obtained laboratory results is higher than labeled data even though their measured protein content is lower than labeled facts.

Data in Table 3 regarding carbohydrate and protein content explained this situation, as all analyzed products had higher CHO content compared to labeled data, while protein drinks had lower measured CHO content than labeled data.

Our results of labeling inaccuracy of sports foods are consistent with the results of a study done on immune-boosting products, which found that out of the 30 dietary supplements evaluated, 17 had inaccurate labels based on product analysis. Of these 17 products, 13 listed ingredients on the label that weren't detected through analysis, while in the other nine products, substances were detected that weren't listed on the label.

Although athletes tend to consume protein-dense sources to increase their muscle mass, protein recovery, or as a snack, the obtained results are controversial to this hypothesis, which leads to uncontrolled weight management as good impairment of their physical performance and failure to reach the target muscle mass due to over or under-consumption of calories and protein. Moreover, there is a possibility of the presence of other ingredients undeclared on the label and contamination by World Anti-Doping Agency (WADA) prohibited substances which result in serious health, performance, and career consequences according to a review of 50 studies, were published in the period between 1996 and 2021, which investigated the presence of unlabeled compounds in more than 3100 dietary supplements and reported that more than one-quarter of the analyzed dietary supplements pose a potential risk of unintentional doping^37^. Furthermore, a study was done in Iran; two anabolic steroids were surveyed in 30 protein-based supplements, showing that 11 of the supplements (36.67%) of the total samples were contaminated with 4-androstenedione^38^.

Surprisingly mean fat content per serving of 30 samples out of 45 samples was higher than the labeled information in the bar, puffs, vegan protein, and protein drinks by 37.8, 32.7, 149.3, and 28.6%, respectively. This may lead to gain undesirable weight, not muscle mass, which is considered adulteration for consumers. Our findings agreed with the hypothesis stated that mean muscle mass increase with high protein consumption, not fat or carbohydrate content. Despite the inaccurate data about protein content among 14 different protein bar products, 80% of sport nutrition sales comes from protein-based products, mainly protein bars and powdered formals^1^.

To the best of our knowledge, this study is the first one that has analyzed the proximate content, including fat, protein, carbohydrates, and total energy, of the available commercialized high-protein sport food products available in the Egyptian market. Interestingly, our data confirmed the inaccuracy of nutritional labeling facts that most gym users and athletes rely on to complete their recommended intake of protein. Our concern about the poor production control of food supplements is not only in Egypt but also in Europe according to a Bulgarian study stated that many researchers support the opinion that there is a real risk for the distribution of low-quality products in countries where the production control is inadequate, and this concern provoked the European Parliament and the Council to undertake a number of legislative initiatives in the field of production and control of food products.

The present study has both strengths and limitations that should be acknowledged. The strengths include the fact that the study is a pioneer in the sports nutrition research area as it is considered the first one which analyzes claimed high protein sport food products available in markets. The present study also has some notable limitations, the laboratory wherein the analyses were conducted lacks formal accreditation; notwithstanding, meticulous annual calibration of the employed instrumentation was undertaken, and deliberate replication of analyses was performed upon the studied samples. These measures collectively served to ascertain the robust reproducibility of the obtained outcomes, also the provided information about fat and protein content reflecting its quantity (content g/serving) only, not quality, such as the quality of analyzed protein (amino acid sequence, plant-based or animal source, amount of Branched Chain Amino Acids (BCAA) particularly leucine and presence or absence of creatine), the quality of fat such as its compositions and components. Also, these products were not screened for the presence of doping substances.

## Conclusion and recommendations

The use of protein supplements is becoming more widespread in the context of athletics, and their promotion has grown recently. Nonetheless, the quality inspection must be done in accordance with the manufacturer's labeling instructions. A database was established regarding proximate analysis as supplemental information by analyzing 45 available protein sports supplements that are currently commercialized in the markets.

To the best of our knowledge, this is the first study that analyzes nutritional facts of protein sports food. Our data indicate that food labels of protein supplements lack accuracy; therefore, our study raises the need for the significant role of those who are considering purchasing or using dietary supplements to assess the rationale for using such products. The studied products involved protein bars, cookies, powder, whey, vegan protein, and drinks. Most of the studied samples had higher total energy (kcal/serving) than reported on labeling data. The increase in energy was a result of elevated content in either carbohydrates or/ and fats, although consumers use these products to get their recommended calories in the form of high protein products.

Lastly, while protein supplements are an adequate tool in the athlete's diet, specialists must consider specific factors, such as those examined in this study, before making any recommendations. Furthermore, supplementation must always be considered in the context of a healthy, diverse, and balanced diet and must never represent a danger to the consumer, who must be properly educated and advised in connection to his aims and requirements.

Our recommendations are the necessity of the establishment of a Council to undertake several legislative initiatives in the field of production and control of sports food products, set more precise quality and safety criteria in future strategies associated with those products, and mandatory nutritional analysis of these products before their release in the market by the accredited laboratories in the Egyptian Universities and Institutes.

## Data Availability

The data used to support the findings of this study can be made available by the corresponding author upon request.
